# d-Serine as the gatekeeper of NMDA receptor activity: implications for the pharmacologic management of anxiety disorders

**DOI:** 10.1038/s41398-020-00870-x

**Published:** 2020-06-09

**Authors:** Herman Wolosker, Darrick T. Balu

**Affiliations:** 1 Department of Biochemistry, Rappaport Faculty of Medicine, Technion-Israel Institute of Technology, Haifa, 31096 Israel; 2 Department of Psychiatry, Harvard Medical School, Boston, MA 02115 USA; 3 Translational Psychiatry Laboratory, McLean Hospital, Belmont, MA 02478 USA

**Keywords:** Molecular neuroscience, Psychiatric disorders

## Abstract

Fear, anxiety, and trauma-related disorders, including post-traumatic stress disorder (PTSD), are quite common and debilitating, with an estimated lifetime prevalence of ~28% in Western populations. They are associated with excessive fear reactions, often including an inability to extinguish learned fear, increased avoidance behavior, as well as altered cognition and mood. There is an extensive literature demonstrating the importance of *N-*methyl-d-aspartate receptor (NMDAR) function in regulating these behaviors. NMDARs require the binding of a co-agonist, d-serine or glycine, at the glycine modulatory site (GMS) to function. d-serine is now garnering attention as the primary NMDAR co-agonist in limbic brain regions implicated in neuropsychiatric disorders. l-serine is synthesized by astrocytes, which is then transported to neurons for conversion to d-serine by serine racemase (SR), a model we term the ‘serine shuttle.’ The neuronally-released d-serine is what regulates NMDAR activity. Our review discusses how the systems that regulate the synaptic availability of d-serine, a critical gatekeeper of NMDAR-dependent activation, could be targeted to improve the pharmacologic management of anxiety-related disorders where the desired outcomes are the facilitation of fear extinction, as well as mood and cognitive enhancement.

Pathological fear and anxiety disorders, including post-traumatic stress disorder (PTSD), which are associated with exaggerated reactions to fearful stimuli and an inability to extinguish learned fear, underlie some of the most common and debilitating psychiatric disorders^1^. The understanding of the neural circuitry and genetics underlying PTSD has rapidly progressed over recent years, and there is great interest in developing novel pharmacologic treatments based on these findings. Human neuroimaging and rodent models have implicated numerous cortical, subcortical, and midbrain regions in producing the symptoms observed in patients with PTSD (Fig. 1a). This disorder is frequently conceptualized as a memory disorder with dysregulated fear learning at the core of many of its signs and symptoms^2^. Three of the most well studied and interconnected brain regions linked to PTSD symptoms are the amygdala, medial prefrontal cortex (mPFC), and hippocampus (HP). In PTSD, there is a failure of top-down cortical inhibition, leading to the reactivation of memories associated with trauma-related thoughts and feelings. Failure of top-down inhibition impairs the ability to extinguish fear^3^, which is the active learning of a new non-threatening association. Thus, previously dangerous stimuli are no longer considered fearful. PTSD patients exhibit deficits in recall of extinction memory and display diminished activation of the mPFC and HP, which correlates with symptom severity and disrupted prefrontal-amygdala functional connectivity^3,4^. In addition, recent evidence suggests that the neurobiological underpinnings related to altered cognition and mood are due to dysfunctions in the hippocampus and amygdala and their ability to regulate PFC top-down control^5^.

**Fig. 1:**
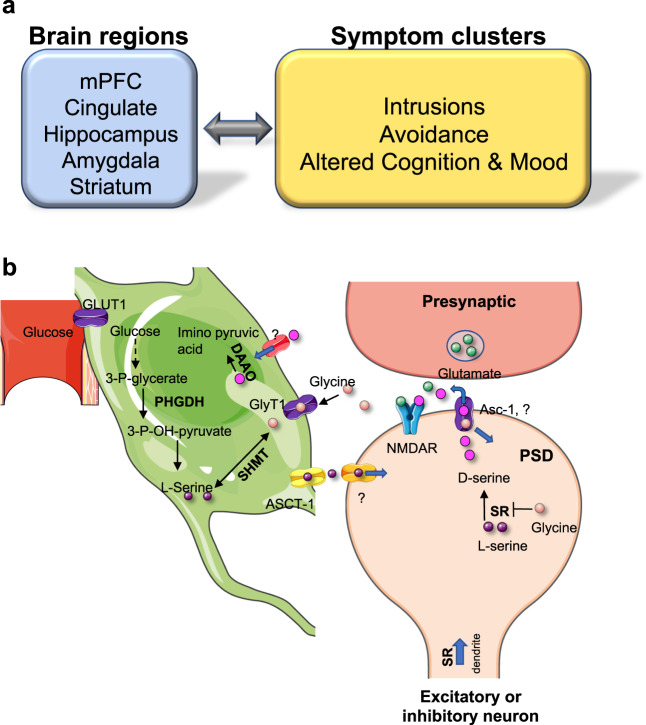
D-serine circuits, sympotom clusters, and the ‘serine shuttle'. **a** Diagram illustrating the human and rodent forebrain regions expressing high levels of serine racemase that are implicated in the major post-traumatic stress disorder symptom clusters. **b** Schematic representation of the serine shuttle in astrocytes and neurons. Glucose is transported from the blood into astrocytes by glucose transporter-1. Most brain l-serine is synthesized *de novo* from the glycolytic intermediate 3-phosphoglycerate, with phosphoglycerate 3-dehydrogenase being the committed step in astrocytic l-serine biosynthesis. l-serine is released from astrocytes by the alanine/serine/cysteine/threonine transporter-1 and is then taken up into neurons via currently unidentified transporter(s), where it is converted to d-serine by the enzyme serine racemase. Serine racemase is strongly inhibited by glycine, which competes for binding with l-serine. Serine racemase and d-serine are concentrated in dendrites and dendritic spines of glutamatergic and GABAergic neurons. d-serine is released from neurons, in part, by the alanine-serine-cysteine-1 transporter, where it binds to the glycine modulatory site on synaptic *N*-methyl-d-aspartate receptors. Glycine also regulates d-serine metabolism by affecting the efficiency of d-serine transport, as it is a high-affinity substrate of the alanine-serine-cysteine-1 transporter and can enhance the release of d-serine by amino acid hetero-exchange. d-serine can be eliminated from the synaptic space by either reuptake into astrocytes where it is catabolized by d-amino acid oxidase or via neuronal serine racemase. Asc-1 alanine-serine-cysteine-1, Slc7a10 solute carrier family 7 member 10, ASCT-1 alanine/serine/cysteine/threonine transporter-1 (Slc1a4; solute carrier Family 1 member 4), DAO d-amino acid oxidase, GLUT1 glucose transporter-1, GlyT-1 glycine transporter-1, mPFC medial prefrontal cortex, NMDAR *N*-methyl-d-aspartate receptor, PHGDH phosphoglycerate 3-dehydrogenase, *PSD* postsynaptic density, *SHMT* serine hydroxymethyltransferase, SR serine racemase, Some clipart in this figure were downloaded from https://smart.servier.com.

Pavlovian fear conditioning is one of the most widely used models for studying emotional memory and associative learning in rodents^6^. The amygdala is a central hub in the emotional learning circuit, integrating sensory information from both cortical and subcortical brain regions related to the conditioning and extinction experience^7^. Although multiple neurotransmitter systems can regulate extinction learning, many studies demonstrate the importance of NMDAR function in extinction learning using antagonists given either systemically or intracranially. In particular, NMDARs within the amygdala, mPFC, and hippocampus are essential for the acquisition and the extinction of fear memories and their associated physiologic symptoms^8^. NMDARs require the binding of a co-agonist, d-serine or glycine, at the glycine modulator site (GMS) to function. d-serine is functionally a more potent activator of synaptic NMDARs than glycine^9^, and mounting evidence suggests that it serves as the major NMDAR co-agonist in limbic brain regions implicated in neuropsychiatric disorders^10^. Finally, clinical evidence suggests that D-cycloserine (DCS), a partial agonist at the NMDAR GMS, is modestly effective at treating patients with anxiety disorders, including PTSD, in conjunction with cognitive behavioral therapy^11,12^. In the following sections, we discuss how the systems that regulate d-serine, a critical gatekeeper of NMDAR-dependent activation, could be targeted to improve the pharmacologic management of anxiety-related disorders where the desired outcomes are the facilitation of fear extinction, as well as mood and cognitive enhancement.

## PTSD symptom domains and brain circuits

Intrusion symptoms in PTSD as defined in the DSM-5, are those in which the traumatic event is persistently re-experienced and can include recurring involuntary intrusive memories and physiological reactivity^13^. Individuals with PTSD often display increased amygdala activity and decreased medial prefrontal cortex (mPFC) activity during symptom provocation studies when compared with controls^14–17^. This suggests that the reactivation of trauma-related memories in PTSD is associated with a failure of top-down cortical inhibition (e.g., from the mPFC) of the reactivation of trauma-related memories^18^. Failure of top-down cortical inhibition might also underlie fear extinction impairments in PTSD^3^.

In classical conditioning, fear conditioning occurs when a neutral cue (a tone or an image) is paired with an intrinsically aversive stimulus, such as electric shock, whereby subsequent presentations of the neutral cue induce a fear response. Fear extinction refers to the gradual reduction of the fear response to a conditioned stimulus when it fails to be reinforced^19^. There is strong evidence that fear extinction involves the formation of a competing new memory that inhibits the fear response rather than an erasure of the original memory^19,20^. However, fear memories may also weaken during recall through a process called reconsolidation^21^. Although individuals with PTSD can encode new fear extinction memories, they do not retain them as well^3,22,23^, suggesting a deficit in fear extinction retention that underlies PTSD symptoms. In PTSD subjects, the size and activity of the ventromedial PFC (vmPFC) is associated with the extent of fear extinction^24^ and the changes to the functional connectivity between the vmPFC and the amygdala^25^. These circuit changes could offer a mechanistic basis for the extinction retention impairments observed in PTSD subjects, since vmPFC-mediated inhibition of the amygdala is thought to be necessary for fear extinction^26^.

Effortful avoidance of distressing trauma-related stimuli is another DSM-5 PTSD symptom domain^13^. Imaging studies suggest that avoidance symptoms and fear circuit activation are closely linked, implicating the anterior cingulate and inferior frontal cortices, as well as hippocampus and amygdala. They also suggest that avoidance is integral to the observed PTSD fear extinction deficits^5^. Since a cue or context that is avoided cannot be extinguished, behavioral therapy approaches for PTSD focus on decreasing avoidance behaviors.

Negative alterations in cognition and mood that begin or worsen after a traumatic event are another criterion in the DSM-5 PTSD diagnosis and include memory deficits and anhedonia symptomatology^13^. Many of these alterations highly overlap with the symptoms of depression. Although preliminary, evidence points towards aberrations in limbic brain regions, particularly the hippocampus and amygdala, and their relationship with top-down PFC control. As the hippocampus is crucial for learning and memory processes, particularly declarative memory^27^, hippocampal dysfunction has been proposed to account for PTSD memory deficits^28^. The hippocampus is involved in the initial storage and integration of aspects of memory during retrieval. A substantial literature, including the largest brain imaging study of PTSD to date, demonstrates reduced hippocampal volume in PTSD patients^29,30^. Individuals with PTSD also exhibit decreased hippocampal activity while taking part in a declarative memory task when compared with trauma-exposed controls without PTSD^31^, as well as decreased hippocampal activity and a failure to recall extinction learning when taking part in a fear conditioning paradigm^3^. Finally, extensive research also shows that individuals with PTSD have deficits in a number of executive function tasks^32^.

## d-Serine mediated nmdar activation and behavior

As described above, the neural circuit abnormalities that contribute to the pathophysiology of PTSD are becoming more well defined. This section will focus on several interconnected limbic brain regions, including the amygdala, hippocampus, and mPFC, for which NMDAR-dependent activation is well-established in mediating behaviors in animal models that are relevant for the symptoms observed in PTSD patients (Table 1). Specifically, we highlight what is known about d-serine dependent NMDAR activation, as both serine racemase (SR) and d-serine are enriched in excitatory and inhibitory neurons of these cortico-limbic brain regions^33–35^.

**Table 1:** Findings supporting the role of d-serine in fear conditioning and anxiety disorders.

Study	Main finding
Miserendino et al.^142^	Amygdalar NMDARs regulate conditioned fear acquisition in rats
Wolosker et al.^143^	The purification, characterization and cloning of serine racemase, the enzyme that synthesizes d-serine
Rodrigues et al.^144^	The GluN2B subunit of amygdalar NMDARs regulate conditioned fear acquisition in rats
Walker et al.^145^	Intra-amygdala d-cycloserine facilitates the acquisition of conditioned fear in rats
Ressler et al.^146^	In controlled trial, systemic d-cycloserine enhances fear extinction in humans with acrophobia
Heresco-Levy et al.^89^	In controlled trial, d-serine treatment reduces symptoms in humans with post-traumatic stress disorder
Balu et al.^41^	Trace fear conditioning impairments in mice lacking serine racemase are restored by systemic treatment with d-serine
Balu et al.^34^	Serine racemase and d-serine are localized to neurons, but not astrocytes, in mouse and human brain
Li et al.^10^	Endogenous d-serine mediates NMDAR function during tonic activation in mouse amygdala
Balu et al.^33^	Serine racemase and d-serine are dynamically regulated by fear conditioning and extinction in the mouse amygdala

*NMDARs* NMDA receptors.

NMDARs are unique compared to other ionotropic glutamate receptors because of their slow deactivation kinetics, high permeability to calcium, and their role as molecular coincidence detectors. Calcium influx through the NMDAR in neurons triggers a cascade of intracellular events that mediate local, acute functional synaptic plasticity and changes in gene expression that further influence synaptic plasticity^36^. In addition to the binding of its agonist glutamate to the GluN2 subunit, NMDAR activation requires postsynaptic depolarization, which relieves the Mg^2+^ blockade of the channel, and the binding of either glycine or d-serine at the GMS on the GluN1 subunit^37,38^. Although the co-agonists glycine and d-serine are present in the extracellular space^37^, the GMS is not saturated in vivo^39^. Importantly, d-serine is functionally more effective than glycine in activating NMDARs and is essential for NMDAR-dependent long-term potentiation (LTP) in numerous adult forebrain regions, including the hippocampus, amygdala, mPFC, and striatum^9,10,40–44^. It should be noted that glycine does serve a role in maintaining NMDAR-dependent plasticity in some adult synapses, such as in the thalamo-lateral amygdala^10^ and medial perforant path-dentate gyrus synapses^9^.

Individuals with PTSD display impairments in the extinction of traumatic memories. Although multiple neurotransmitter systems can regulate fear extinction learning, there is a very extensive literature demonstrating the importance of NMDAR function in extinction learning using antagonists given either systemically or intracranially. In particular, NMDARs within the amygdala and mPFC are essential for the acquisition and the extinction of fear memories and their associated physiologic symptoms^8^. NMDAR function in the basolateral amygdala (BLA) is also critical for extinguishing conditioned fear responses, as many studies have shown that NMDAR antagonists delivered into the BLA impair extinction retrieval, while the infusion of DCS or d-serine into the BLA enhances extinction retrieval^11^. The extinction of conditioned fear memory also depends on the mPFC, and in particular the infralimbic (IL) division, which sends very strong projections to the BLA^8,45–48^. Extinction training induces NMDAR-dependent plasticity and increases burst firing in IL neurons, which stabilizes fear extinction memory^49^. Ligands of the NMDAR GMS, such as d-serine, are required for extinction learning. We have shown that SR levels are dynamically up-regulated in the hippocampus, amygdala, and mPFC after fear memory extinction^33^. Mice that lack SR and have 90% lower d-serine, display impaired post-retrieval extinction of contextual fear memory that can be restored by d-serine administration, implicating a role for d-serine in the reconsolidation process^50^. Exogenous d-serine or DCS administration facilitates the acquisition and retention of fear extinction^11,33,51^, while d-serine also facilitates the extinction of drug seeking behavior^52^. Clinical evidence also suggests that DCS is modestly effective at treating patients with anxiety disorders, including PTSD, in conjunction with cognitive behavioral therapy^11,12^. In addition, a recent placebo-controlled, double-blind, three-day fear conditioning and delayed extinction fMRI study in healthy participants, found that DCS enhanced extinction consolidation, as reflected by reduced arousal ratings and activation of brain regions that mediate defensive reactions^53^. Interestingly, findings in mice suggest that DCS may also serve as a precursor to d-serine in the brain^54^. Finally, there is genetic evidence linking d-serine with PTSD. A single nucleotide polymorphism (SNP; rs4523957) within the human serine racemase (*SRR*) gene previously associated with other disorders^55–57^, is a functional eQTL at the level of regulating SR mRNA expression in *post-mortem* human brain and is associated with PTSD^33^ in a highly traumatized civilian population^58,59^.

While extinction of classical fear conditioning (e.g., inhibitory and safety learning) relies on lower limbic implicit memory systems and processing of negative valence as a function of threat expectancy^60^, fear extinction of hippocampus-dependent learning requires more complex, higher order associative learning processes^61^. As such, episodic and semantic memory systems are predominantly prefrontal cortical and hippocampal/temporal, and thus likely engage different memory systems than those used for processing implicit, subcortical memory associations^62^. Since NMDAR antagonism can produce discrete impairments in episodic and semantic memory^63^, it is possible that increasing endogenous d-serine levels to increase NMDAR activity could facilitate the extinction of fear memories that engage higher-order processing.

Emerging literature describes the brain circuits engaged in mediating passive (freezing) and active avoidance strategies (e.g., escaping to a safe chamber) when an animals are presented with threat-associated stimuli^64^. Rodent lesion studies indicate that passive freezing is mediated by signals transmitted from the lateral amygdala to the central amygdala and then to the periaqueductal gray^65^. d-serine administration enhances memory extinction in an inhibitory avoidance task^66,67^, potentially through increasing GluA2-containing AMPA receptor endocytosis^67^.

Individuals with PTSD exhibit negative alterations in cognition and mood, such as anxiety and social withdrawal, that rely on proper NMDAR function and begin or worsen after a traumatic event. Numerous studies have demonstrated the importance of endogenous d-serine in mediating NMDAR activation for contextual and working memory in rodents^41,42,50,68–70^. Furthermore, endogenous d-serine is important for maintaining proper dendritic spine density and dendritic arborization of excitatory neurons and promotes the proliferation and survival of adult-born hippocampal neurons^41,71–76^. Preclinical studies indicate that d-serine or DCS administration can normalize behaviors used as models for anxiety and depression, as well as social memory and social interaction deficits, in genetic and pharmacologic rodent models^77–80^. It should be noted that small clinical studies suggest that NMDAR antagonism either with ketamine^81,82^ or Ifenprodil (GluN2B-specififc)^83,84^ had beneficial effects in treating depressive symptoms in PTSD patients and flashbacks of adolescent female PTSD patients with a history of abuse, respectively, through undefined mechanisms. Furthermore, the role of d-serine in neuroplasticity would presumably be beneficial to PTSD patients given that in rodents, stress, a key environmental risk factor for PTSD, reduces hippocampal volume, adult neurogenesis, dendritic complexity, and spine density^85^. These findings in rodents comport with neuroimaging studies demonstrating that patients with PTSD have reduced volumes of the hippocampal and prefrontal brain regions^30,86,87^. Finally, clinical studies demonstrate that GMS agonists, including d-serine, can improve cognition in healthy participants and patients with neuropsychiatric disorders^88–91^. It should be noted that the majority of these clinical studies had small sample sizes and included subjects primarily with schizophrenia, PTSD, or dementia. However, these proof-of-concept clinical trials do provide evidence supporting the use of NMDAR GMS agonists to improve mood and cognition.

## d-Serine metabolism, uptake, and release

### d-Serine production and localization

d-Serine synthesis is carried out by SR, which catalyzes the racemization of l-serine into d-serine^92^. A distinctive feature of SR is the catalysis of a parallel reaction consisting of the α, β-elimination of water from l-serine and production of pyruvate and ammonia, suggesting that SR also has a catabolic function^93^. d-Serine and SR were initially thought to be exclusively present in astrocytes, leading to a series of studies that investigated the effects of “glial” d-serine^40,94,95^. However, as recently reviewed in detail^96,97^, studies purporting d-serine as a “gliotransmitter” lack the proper controls to ensure that the effects observed in NMDAR physiology are due to glial d-serine, such as the use of cell-selective SR-KO mice. The generation of more selective antibodies to SR and better techniques to detect d-serine^98^, along with the use of SR-KO mice as controls for immunostaining^35,99,100^ demonstrated that SR is preferentially expressed in neurons and d-serine having a neuronal origin. Cell-selective deletion of SR indicates that glutamatergic neurons are the primary site of d-serine synthesis^99,101^, whereas the deletion of SR from astroglia had little effect on brain d-serine^99^. NMDAR-dependent hippocampal plasticity is impaired in vitro and in vivo by the elimination of SR from glutamatergic neurons, while the deletion of astrocytic SR had no effect, indicating that astrocytic d-serine does not play a role in synaptic plasticity under normal conditions^99,102^.

### The serine shuttle

A constant supply of l-serine is critical for d-serine synthesis, as the intracellular levels of l-serine (~1 mM) are one order of magnitude below the apparent affinity of SR to l-serine^93^. Although l-serine is a non-essential amino acid, most brain l-serine is synthesized from the glycolytic intermediate 3-phosphoglycerate by the sequential actions of three astrocyte-specific enzymes, phosphoglycerate dehydrogenase (Phgdh), phosphoserine aminotransferase 1 (Psat-1), and phosphoserine phosphatase (Psph)^103^. Mutations in Phgdh, the committed step in astrocytic l-serine biosynthesis, cause microcephaly and severe neurodevelopmental deficits in humans attributable to deficits in brain l-serine^104,105^. In agreement with human genetic studies, the astrocytic knockout of Phgdh decreases brain l-serine and d-serine in mice^106^. This is associated with a decrease in the neuronal staining of d-serine^100^, suggesting the existence of a “serine shuttle”, whereby astrocytic l-serine shuttles to neurons to sustain neuronal synthesis of d-serine (Fig. 1b). Pharmacological inhibition of Phgdh in acute brain slices impairs NMDAR-dependent hippocampal functional plasticity, without changing basal neurotransmission^101^, supporting the notion that the serine shuttle is essential for NMDAR activation^107^.

The ASCT1 (Slc1a4) transporter mediates the export of l-serine and other neutral amino acids from astrocytes^69,108^. Mice with targeted deletion of ASCT1 have lower brain d-serine, associated with a reduction in hippocampal volume, impairments in spatial memory, and motor dysfunction^69^. Similar to patients with mutations in Phgdh, children with loss-of-function mutations in ASCT1 display microcephaly and severe neurodevelopmental deficits^109–111^, highlighting the role of astrocytic l-serine in neurodevelopment.

Hitherto unidentified transporters mediate the uptake of astrocyte-derived l-serine in neurons. Possible candidates include the Asc-1 (Slc7a10) neutral amino acid antiporter^112^, system A (Slc38 family) transporters^113^, or system L (Slc7 family) antiporters with broad specificity to zwitterionic amino acids^114^. Once synthesized by SR, neuronal d-serine is released, at least in part, through Asc-1, which mediates d-serine efflux by exchange with extracellular neutral amino acids and/or facilitated diffusion^115,116^. Targeted deletion or pharmacological inhibition of Asc-1 decreases the extracellular levels of d-serine^117^ and impairs NMDAR-dependent synaptic plasticity in hippocampal Schaffer collaterals-CA1 synapses^116^. Conversely, activation of the Asc-1 antiporter by increasing the levels of extracellular Asc-1 substrates enhances the d-serine release and promotes NMDAR synaptic activation^115^. Asc-1 also uses glycine as substrate. Asc-1-KO mice display lower brain glycine and hyperekplexia due to the impairment of glycinergic inhibitory transmission that is preventable by administering glycine to the mice^112^. These observations indicate that increasing the hetero-exchange activity of Asc-1 by selective substrates provides a strategy to increase the availability of NMDAR co-agonists.

### Regulation of d-serine production at the postsynaptic site

Different from classical transmitters, d-serine appears to be mainly produced at postsynaptic sites. Subcellular fractionation demonstrated co-purification of SR with detergent-resistant postsynaptic density membranes^118^. SR is localized to neurons^34,35^, where it is enriched at dendritic spines^74^. Furthermore, SR contains a PDZ binding region at its C-terminus and associates with several postsynaptic-enriched proteins, such as glutamate receptor interacting protein 1 (Grip-1)^119^, discs large MAGUK scaffold protein 3 (SAP-102)^120^, stargazin^120^, discs large MAGUK scaffold protein 4 (PSD-95)^74,120^, Disrupted in schizophrenia 1 (DISC1)^121^, and protein interacting with PRKCA 1 (PICK1)^122^. The proximity of SR to the postsynaptic sites provides a mechanism for local activation of synaptic NMDARs. Partial saturation of synaptic NMDARs by tonic d-serine release would allow immediate activation of NMDARs upon glutamate binding.

α-amino-3-hydroxy-5-methyl-4-isoxazolepropionic acid receptor (AMPAR) stimulation has indirect effects on SR activity. In vitro evidence suggests that SR forms a quaternary complex with PSD-95, stargazin, and AMPARs, which partially inhibits the synthesis of d-serine. AMPAR activation leads to the dissociation of SR from stargazin and increases SR activity, providing a crosstalk between AMPAR and NMDAR activities that may play a role in synaptic homeostasis^120^.

NMDAR activation leads to a cascade of events that inhibits SR activity by different mechanisms, providing a feedback regulation of NMDAR activity. NMDAR activation triggers the production of nitric oxide (NO) by nitric oxide synthase, leading to the S-nitrosylation of SR and inhibition of d-serine synthesis^123^. NMDAR stimulation also elicits the translocation of SR from the cytosol to membranes and the cell nucleus, where the enzyme is mostly inactive^118,124^. Feedback inhibition of SR likely serves to prevent NMDAR neurotoxicity under situations of increased neuronal activity. Conversely, blockade of NMDARs by chronic MK-801 administration increases SR expression in the brain^125^, providing another connection between NMDAR activity and d-serine production.

SR levels are also regulated by the proteasomal system^126^. DISC1 binds to and stabilizes SR by decreasing its degradation through the proteasome^121^. DISC1 truncation segregates with schizophrenia and other psychiatric conditions in a Scottish family^127^. Mice expressing mutant DISC1 exhibit lower SR and d-serine levels that are associated with behavioral alterations^121^. In addition to feedback regulation, SR is strongly inhibited by glycine, which competes with l-serine for binding to SR^128,129^. Injection of glycine leads to a decrease in the extracellular levels of d-serine in vivo, indicating their metabolism is connected^101^. Inhibition of SR by glycine ensures that little d-serine will be produced in the brainstem and spinal cord, where glycine is the major inhibitory neurotransmitter^130^. Glycine also regulates d-serine metabolism by affecting the efficiency of d-serine transport. Like d-serine, glycine is a high-affinity substrate of the Asc-1 transporter, and it enhances the release of d-serine via Asc-1 by amino acid hetero-exchange^101^. The dual role of glycine in regulating d-serine metabolism is puzzling, and their regional variation and distinct half-lives provide a plethora of mechanisms to fine-tune NMDAR activity.

### d-Serine catabolism

In the forebrain, d-serine has a half-life of 16.9 h^131^, indicating slow metabolism. In comparison, metabolic labeling indicates that GABA and glutamate half-lives are around 30 min^132^. Although d-serine can be degraded by d-amino acid oxidase (DAO) in peroxisomes, this enzyme is mostly restricted to the cerebellum, brainstem, and spinal cord^133^. Mice expressing a catalytically-inactive DAO enzyme display no changes in cortical d-serine, indicating that DAO does not play a significant metabolic role in the adult forebrain^134^. Human DAO expression is more widespread in forebrain regions, but the very low affinity for its cofactor FAD suggests this enzyme does not efficiently degrade d-serine in humans^135^. Another possible catabolic route for d-serine is the SR enzyme itself, which can degrade d-serine into pyruvate and ammonia by the α,β-elimination reaction^93^. However, although this pathway can play a role in limiting the build-up of d-serine in forebrain regions, the rate of conversion of l-serine into d-serine is faster than the α,β-elimination with d-serine^136^, indicating that the d-serine synthesis is the preferential reaction of SR.

Transport mechanisms to remove d-serine from the synapse are not as efficient as with classical transmitters. d-serine reuptake systems are not stereoselective and display only moderate to low-affinity for the d-enantiomer^137^. Therefore, we predict that d-serine will remain at the synapse for prolonged periods of time and will be functionally more effective than glycine, as powerful glycine transporters (e.g., glycine transporter-1; GlyT1), limit glycine access to synaptic NMDARs^39,138^. In contrast to classical transmitters, neuronal depolarization has only modest effects on d-serine release^139^. In this framework, we propose that d-serine works as an NMDAR gatekeeper that is tonically released at postsynaptic sites. The selective action of d-serine at NMDARs and its role in regulating behavior provides an opportunity to develop drugs that will gently affect NMDAR function by affecting the basal occupancy of the receptor.

## Conclusions and future directions

d-serine is a dynamic gatekeeper of NMDAR function in forebrain regions that are implicated in the pathophysiology of fear and anxiety-related disorders. We highlight the potential utility of d-serine or molecules that augment d-serine availability as a means to enhance extinction learning, as well as improving cognition and mood. The latter strategy could be accomplished by increasing release or blocking reuptake via the aforementioned transporters or inhibiting the breakdown of d-serine by DAO (Table 2). While most of the properties of d-serine metabolism were characterized in the hippocampus and neocortex, it is likely that they are also conserved in the amygdala, as the expression of SR and other components of the serine shuttle are widespread throughout the brain. It will also be useful to test pharmacologic tools that augment d-serine mediated NMDAR-activation using rodent models of impaired extinction, which aim to recapitulate the aberrant extinction learning observed in PTSD patients^140^. In addition, we propose the novel idea that d-serine is not a pre-synaptically released co-agonist, but a postsynaptically released “autocrine” molecule. Thus, the receptive neuron, not the glutamatergic input, determines NMDAR functionality. We hope this review helps to spur new lines of investigation into the mechanisms that regulate d-serine availability across brain regions and the relative contribution of GMS agonists at NMDARs on excitatory versus inhibitory neurons. We and others have shown strong d-serine immunoreactivity in several classes of GABAergic interneurons in the hippocampus, amygdala, cortex, and striatum^33–35,74^. Little is known about the regulation of SR in inhibitory neurons, but the postsynaptic localization of SR suggests that d-serine could also play an “autocrine” role in activating NMDARs on GABAergic neurons. Such findings could potentially help identify novel therapeutic targets to enhance d-serine mediated NMDAR function. A need for a deeper understanding of d-serine mediated NMDAR activation is highlighted by the modest success of DCS, a *partial agonist* at the GMS site, in augmenting exposure therapy outcomes in patients with anxiety disorders and PTSD^12,141^.

**Table 2:** Putative novel pharmacologic strategies to increase d-serine mediated NMDA receptor transmission as a means to treat anxiety-related disorders.

Target	Function	Pharmacologic manipulation
Asc-1 (Slc7a10)	d-Serine/amino acid exchanger	Activator^116,147^
ASCT1 (Slc1a4)	d-Serine/amino acid exchanger	Activator^69^
DAAO	Enzyme that breaks down d-serine	Inhibitor^148–150^
Serine racemase	Enzyme that converts l-serine to d-serine	Activator^151^

*Asc1* alanine-serine-cysteine-1 transporter, *ASCT1* alanine/serine/cysteine/threonine transporter-1, *DAAO*d-amino acid oxidase.

## References

[1] Kessler RC (2005). Lifetime prevalence and age-of-onset distributions of DSM-IV disorders in the National Comorbidity Survey Replication. Arch. Gen. Psychiatry.

[2] Jovanovic T, Ressler KJ (2010). How the neurocircuitry and genetics of fear inhibition may inform our understanding of PTSD. Am. J. Psychiatry.

[3] Milad MR (2009). Neurobiological basis of failure to recall extinction memory in posttraumatic stress disorder. Biol. Psychiatry.

[4] Sheynin J, Liberzon I (2017). Circuit dysregulation and circuit-based treatments in posttraumatic stress disorder. Neurosci. Lett..

[5] Fenster RJ (2018). Brain circuit dysfunction in post-traumatic stress disorder: from mouse to man. Nat. Rev. Neurosci..

[6] LeDoux JE (2000). Emotion circuits in the brain. Annu. Rev. Neurosci..

[7] Janak PH, Tye KM (2015). From circuits to behaviour in the amygdala. Nature.

[8] Tovote P, Fadok JP, Luthi A (2015). Neuronal circuits for fear and anxiety. Nat. Rev. Neurosci..

[9] Le Bail M (2015). Identity of the NMDA receptor coagonist is synapse specific and developmentally regulated in the hippocampus. Proc. Natl Acad. Sci. USA.

[10] Li Y (2013). Identity of endogenous NMDAR glycine site agonist in amygdala is determined by synaptic activity level. Nat. Commun..

[11] Singewald N (2015). Pharmacology of cognitive enhancers for exposure-based therapy of fear, anxiety and trauma-related disorders. Pharm. Ther..

[12] Mataix-Cols D (2017). D-cycloserine augmentation of exposure-based cognitive behavior therapy for anxiety, obsessive-compulsive, and posttraumatic stress disorders: a systematic review and meta-analysis of individual participant Data. JAMA Psychiatry.

[13] American Psychiatric Association. *Diagnostic and Statistical Manual of Mental Disorders (DSM-5)* (ed American Psychiatric Association) (APA Publishing, 2013).

[14] Bremner JD (1999). Neural correlates of memories of childhood sexual abuse in women with and without posttraumatic stress disorder. Am. J. Psychiatry.

[15] Lanius RA (2001). Neural correlates of traumatic memories in posttraumatic stress disorder: a functional MRI investigation. Am. J. Psychiatry.

[16] Rauch SL (1996). A symptom provocation study of posttraumatic stress disorder using positron emission tomography and script-driven imagery. Arch. Gen. Psychiatry.

[17] Shin LM (2004). Regional cerebral blood flow in the amygdala and medial prefrontal cortex during traumatic imagery in male and female Vietnam veterans with PTSD. Arch. Gen. Psychiatry.

[18] Lanius RA (2010). Emotion modulation in PTSD: Clinical and neurobiological evidence for a dissociative subtype. Am. J. Psychiatry.

[19] Milad MR, Quirk GJ (2012). Fear extinction as a model for translational neuroscience: ten years of progress. Annu Rev. Psychol..

[20] Rescorla RA, Heth CD (1975). Reinstatement of fear to an extinguished conditioned stimulus. J. Exp. Psychol. Anim. Behav. Process.

[21] Kroes MC (2016). Translational approaches targeting reconsolidation. Curr. Top. Behav. Neurosci..

[22] Garfinkel SN (2014). Impaired contextual modulation of memories in PTSD: an fMRI and psychophysiological study of extinction retention and fear renewal. J. Neurosci..

[23] Norrholm SD (2011). Fear extinction in traumatized civilians with posttraumatic stress disorder: relation to symptom severity. Biol. Psychiatry.

[24] Rauch SL (2003). Selectively reduced regional cortical volumes in post-traumatic stress disorder. Neuroreport.

[25] Stevens JS (2013). Disrupted amygdala-prefrontal functional connectivity in civilian women with posttraumatic stress disorder. J. Psychiatr. Res..

[26] Phelps EA (2004). Extinction learning in humans: role of the amygdala and vmPFC. Neuron.

[27] Squire LR, Zola-Morgan S (1991). The medial temporal lobe memory system. Science.

[28] Elzinga BM, Bremner JD (2002). Are the neural substrates of memory the final common pathway in posttraumatic stress disorder (PTSD)?. J. Affect Disord..

[29] Logue MW (2018). Smaller hippocampal volume in posttraumatic stress disorder: a multisite enigma-pgc study: subcortical volumetry results from posttraumatic stress disorder consortia. Biol. Psychiatry.

[30] Pitman RK (2012). Biological studies of post-traumatic stress disorder. Nat. Rev. Neurosci..

[31] Bremner JD (2003). MRI and PET study of deficits in hippocampal structure and function in women with childhood sexual abuse and posttraumatic stress disorder. Am. J. Psychiatry.

[32] Polak AR (2012). The role of executive function in posttraumatic stress disorder: a systematic review. J. Affect Disord..

[33] Balu DT (2018). Serine Racemase and D-serine in the amygdala are dynamically involved in fear learning. Biol. Psychiatry.

[34] Balu DT (2014). D-serine and serine racemase are localized to neurons in the adult mouse and human forebrain. Cell Mol. Neurobiol..

[35] Miya K (2008). Serine racemase is predominantly localized in neurons in mouse brain. J. Comp. Neurol..

[36] Greer PL, Greenberg ME (2008). From synapse to nucleus: calcium-dependent gene transcription in the control of synapse development and function. Neuron.

[37] Johnson JW, Ascher P (1987). Glycine potentiates the NMDA response in cultured mouse brain neurons. Nature.

[38] Kleckner NW, Dingledine R (1988). Requirement for glycine in activation of NMDA-receptors expressed in Xenopus oocytes. Science.

[39] Bergeron R (1998). Modulation of N-methyl-D-aspartate receptor function by glycine transport. Proc. Natl Acad. Sci. U.S.A..

[40] Papouin T (2012). Synaptic and extrasynaptic NMDA receptors are gated by different endogenous coagonists. Cell.

[41] Balu DT (2013). Multiple risk pathways for schizophrenia converge in serine racemase knockout mice, a mouse model of NMDA receptor hypofunction. Proc. Natl Acad. Sci. U.S.A..

[42] Basu AC (2009). Targeted disruption of serine racemase affects glutamatergic neurotransmission and behavior. Mol. Psychiatry.

[43] Curcio L (2013). Reduced D-serine levels in the nucleus accumbens of cocaine-treated rats hinder the induction of NMDA receptor-dependent synaptic plasticity. Brain.

[44] Fossat P (2012). Glial D-serine gates NMDA receptors at excitatory synapses in prefrontal cortex. Cereb. cortex.

[45] Amano T, Unal CT, Pare D (2010). Synaptic correlates of fear extinction in the amygdala. Nat. Neurosci..

[46] Milad MR, Quirk GJ (2002). Neurons in medial prefrontal cortex signal memory for fear extinction. Nature.

[47] Senn V (2014). Long-range connectivity defines behavioral specificity of amygdala neurons. Neuron.

[48] Quirk GJ (2000). The role of ventromedial prefrontal cortex in the recovery of extinguished fear. J. Neurosci..

[49] Burgos-Robles A (2007). Consolidation of fear extinction requires NMDA receptor-dependent bursting in the ventromedial prefrontal cortex. Neuron.

[50] Inoue R (2018). Dissociated Role of D-Serine in Extinction During Consolidation vs. Reconsolidation of Context Conditioned Fear. Front Mol. Neurosci..

[51] Matsuda S (2010). d-serine enhances extinction of auditory cued fear conditioning via ERK1/2 phosphorylation in mice. Prog. Neuropsychopharmacol. Biol. Psychiatry.

[52] Hammond S (2013). D-Serine facilitates the effectiveness of extinction to reduce drug-primed reinstatement of cocaine-induced conditioned place preference. Neuropharmacology.

[53] Ebrahimi C (2019). Augmenting extinction learning with D-cycloserine reduces return of fear: a randomized, placebo-controlled fMRI study. Neuropsychopharmacology.

[54] Horio M, Mori H, Hashimoto K (2013). Is D-cycloserine a prodrug for D-serine in the brain?. Biol. Psychiatry.

[55] Shimasaki A (2014). A genetic variant in 12q13, a possible risk factor for bipolar disorder, is associated with depressive state, accounting for stressful life events. PLoS One.

[56] Van der Auwera S (2016). The inverse link between genetic risk for schizophrenia and migraine through NMDA (N-methyl-D-aspartate) receptor activation via D-serine. Eur. Neuropsychopharmacol..

[57] Zhang S (2014). Association of serine racemase gene variants with type 2 diabetes in the Chinese Han population. J. Diabetes Investig..

[58] Andero R (2013). Amygdala-dependent fear is regulated by Oprl1 in mice and humans with PTSD. Sci. Transl. Med..

[59] Gillespie CF (2009). Trauma exposure and stress-related disorders in inner city primary care patients. Gen. Hosp. Psychiatry.

[60] Craske MG, Hermans D, Vervliet B (2018). State-of-the-art and future directions for extinction as a translational model for fear and anxiety. Philos. Trans. R. Soc. Lond. B. Biol. Sci..

[61] Otto MW (2016). Enhancement of psychosocial treatment with d-cycloserine: models, moderators, and future directions. Biol. Psychiatry.

[62] Radulovic J, Ren LY, Gao C (2019). N-Methyl D-aspartate receptor subunit signaling in fear extinction. Psychopharmacology.

[63] Morgan CJ, Curran HV (2006). Acute and chronic effects of ketamine upon human memory: a review. Psychopharmacology.

[64] LeDoux JE (2017). The birth, death and resurrection of avoidance: a reconceptualization of a troubled paradigm. Mol. Psychiatry.

[65] Choi JS, Cain CK, LeDoux JE (2010). The role of amygdala nuclei in the expression of auditory signaled two-way active avoidance in rats. Learn Mem..

[66] Fiorenza NG (2012). Modulation of the extinction of two different fear-motivated tasks in three distinct brain areas. Behav. Brain Res..

[67] Bai Y (2014). D-serine enhances fear extinction by increasing GluA2-containing AMPA receptor endocytosis. Behav. Brain Res..

[68] DeVito LM (2011). Serine racemase deletion disrupts memory for order and alters cortical dendritic morphology. Genes Brain Behav..

[69] Kaplan E (2018). ASCT1 (Slc1a4) transporter is a physiologic regulator of brain d-serine and neurodevelopment. Proc. Natl Acad. Sci. U.S.A..

[70] Balu DT (2016). An mGlu5-Positive Allosteric Modulator Rescues the Neuroplasticity Deficits in a Genetic Model of NMDA Receptor Hypofunction in Schizophrenia. Neuropsychopharmacology.

[71] Balu DT (2012). The NMDA receptor co-agonists, d-serine and glycine, regulate neuronal dendritic architecture in the somatosensory cortex. Neurobiol. Dis..

[72] Balu DT, Coyle JT (2012). Neuronal d-serine regulates dendritic architecture in the somatosensory cortex. Neurosci. Lett..

[73] Balu DT, Coyle JT (2014). Chronic D-serine reverses arc expression and partially rescues dendritic abnormalities in a mouse model of NMDA receptor hypofunction. Neurochem. Int..

[74] Lin H (2016). D-Serine and Serine Racemase Are Associated with PSD-95 and Glutamatergic Synapse Stability. Front. Cell Neurosci..

[75] Sultan S (2013). D-serine increases adult hippocampal neurogenesis. Front. Neurosci..

[76] Sultan S (2015). Synaptic integration of adult-born hippocampal neurons is locally controlled by astrocytes. Neuron.

[77] Terrillion CE (2017). DISC1 in astrocytes influences adult neurogenesis and hippocampus-dependent behaviors in mice. Neuropsychopharmacology.

[78] Zoicas I, Kornhuber J (2019). The role of the N-methyl-D-aspartate receptors in social behavior in rodents. Int. J. Mol. Sci..

[79] Malkesman O (2012). Acute D-serine treatment produces antidepressant-like effects in rodents. Int. J. Neuropsychopharmacol..

[80] Wang J (2017). Epigenetic activation of ASCT2 in the hippocampus contributes to depression-like behavior by regulating D-serine in mice. Front. Mol. Neurosci..

[81] Feder A (2014). Efficacy of intravenous ketamine for treatment of chronic posttraumatic stress disorder: a randomized clinical trial. JAMA Psychiatry.

[82] Hartberg J, Garrett-Walcott S, De Gioannis A (2018). Impact of oral ketamine augmentation on hospital admissions in treatment-resistant depression and PTSD: a retrospective study. Psychopharmacology.

[83] Kishimoto A (2012). Ifenprodil for the treatment of flashbacks in female posttraumatic stress disorder patients with a history of childhood sexual abuse. Biol. Psychiatry.

[84] Sasaki T (2013). Ifenprodil for the treatment of flashbacks in adolescent female posttraumatic stress disorder patients with a history of abuse. Psychother. Psychosom..

[85] McEwen BS, Nasca C, Gray JD (2016). Stress effects on neuronal structure: hippocampus, amygdala, and prefrontal cortex. Neuropsychopharmacology.

[86] Kremen WS (2012). Twin studies of posttraumatic stress disorder: differentiating vulnerability factors from sequelae. Neuropharmacology.

[87] Woon FL, Sood S, Hedges DW (2010). Hippocampal volume deficits associated with exposure to psychological trauma and posttraumatic stress disorder in adults: a meta-analysis. Prog. Neuropsychopharmacol. Biol. Psychiatry.

[88] Peyrovian B (2019). The glycine site of NMDA receptors: A target for cognitive enhancement in psychiatric disorders. Prog. Neuropsychopharmacol. Biol. Psychiatry.

[89] Heresco-Levy U (2009). Pilot controlled trial of D-serine for the treatment of post-traumatic stress disorder. Int J. Neuropsychopharmacol..

[90] Hashimoto K (2019). Genomic triplication of the glycine decarboxylase gene and N-methyl-d-aspartate receptor hypofunction: improvement by glycine and D-cycloserine. Biol. Psychiatry.

[91] Bodkin JA (2019). Targeted treatment of individuals with psychosis carrying a copy number variant containing a genomic triplication of the glycine decarboxylase gene. Biol. Psychiatry.

[92] Wolosker H (1999). Purification of serine racemase: biosynthesis of the neuromodulator D- serine. Proc. Natl Acad. Sci. U.S.A..

[93] Foltyn VN (2005). Serine racemase modulates intracellular D-serine levels through an alpha,beta-elimination activity. J. Biol. Chem..

[94] Panatier A (2006). Glia-derived D-serine controls NMDA receptor activity and synaptic memory. Cell.

[95] Henneberger C (2010). Long-term potentiation depends on release of D-serine from astrocytes. Nature.

[96] Wolosker H, Balu DT, Coyle JT (2016). The rise and fall of the D-serine-mediated gliotransmission hypothesis. Trends Neurosci..

[97] Wolosker H, Balu DT, Coyle JT (2017). Astroglial versus neuronal d-serine: check your controls!. Trends Neurosci..

[98] Kartvelishvily E (2006). Neuron-derived D-serine release provides a novel means to activate N-methyl-D-aspartate receptors. J. Biol. Chem..

[99] Benneyworth MA (2012). Cell selective conditional null mutations of serine racemase demonstrate a predominate localization in cortical glutamatergic neurons. Cell Mol. Neurobiol..

[100] Ehmsen JT (2013). D-serine in glia and neurons derives from 3-phosphoglycerate dehydrogenase. J. Neurosci.: Off. J. Soc. Neurosci..

[101] Neame S (2019). The NMDA receptor activation by d-serine and glycine is controlled by an astrocytic Phgdh-dependent serine shuttle. Proc. Natl Acad. Sci. U.S.A..

[102] Perez EJ (2017). Enhanced astrocytic d-serine underlies synaptic damage after traumatic brain injury. J. Clin. Investig..

[103] Yamasaki M (2001). 3-Phosphoglycerate dehydrogenase, a key enzyme for l-serine biosynthesis, is preferentially expressed in the radial glia/astrocyte lineage and olfactory ensheathing glia in the mouse brain. J. Neurosci..

[104] de Koning TJ (2004). Prenatal and early postnatal treatment in 3-phosphoglycerate-dehydrogenase deficiency. Lancet.

[105] Tabatabaie L (2010). L-serine synthesis in the central nervous system: a review on serine deficiency disorders. Mol. Genet. Metab..

[106] Yang JH (2010). Brain-specific Phgdh deletion reveals a pivotal role for L-serine biosynthesis in controlling the level of D-serine, an N-methyl-D-aspartate receptor co-agonist, in adult brain. J. Biol. Chem..

[107] Wolosker H, Radzishevsky I (2013). The serine shuttle between glia and neurons: Implications for neurotransmission and neurodegeneration. Biochem. Soc. Trans..

[108] Sakai K (2003). Neutral amino acid transporter ASCT1 is preferentially expressed in L-Ser-synthetic/storing glial cells in the mouse brain with transient expression in developing capillaries. The. J. Neurosci.: Off. J. Soc. Neurosci..

[109] Heimer G (2015). SLC1A4 mutations cause a novel disorder of intellectual disability, progressive microcephaly, spasticity and thin corpus callosum. Clin. Genet..

[110] Srour M (2015). A homozygous mutation in SLC1A4 in siblings with severe intellectual disability and microcephaly. Clin. Genet..

[111] Damseh N (2015). Mutations in SLC1A4, encoding the brain serine transporter, are associated with developmental delay, microcephaly and hypomyelination. J. Med. Genet..

[112] Safory H (2015). The alanine-serine-cysteine-1 (Asc-1) transporter controls glycine levels in the brain and is required for glycinergic inhibitory transmission. EMBO Rep..

[113] Broer S (2014). The SLC38 family of sodium-amino acid co-transporters. Pflug. Arch. Eur. J. Physiol..

[114] Pineda M (1999). Identification of a membrane protein, LAT-2, that Co-expresses with 4F2 heavy chain, an L-type amino acid transport activity with broad specificity for small and large zwitterionic amino acids. J. Biol. Chem..

[115] Rosenberg D (2013). Neuronal D-serine and glycine release via the Asc-1 transporter regulates NMDA receptor-dependent synaptic activity. The. J. Neurosci..

[116] Sason H (2017). Asc-1 transporter regulation of synaptic activity via the tonic release of d-serine in the forebrain. Cereb. Cortex.

[117] Sakimura K (2016). A novel Na(+) -Independent alanine-serine-cysteine transporter 1 inhibitor inhibits both influx and efflux of D-Serine. J. Neurosci. Res..

[118] Balan L (2009). Feedback inactivation of D-serine synthesis by NMDA receptor-elicited translocation of serine racemase to the membrane. Proc. Natl Acad. Sci. U.S.A..

[119] Kim PM (2005). Serine racemase: activation by glutamate neurotransmission via glutamate receptor interacting protein and mediation of neuronal migration. Proc. Natl Acad. Sci. U.S.A..

[120] Ma TM (2014). Serine racemase regulated by binding to stargazin and PSD-95: potential N-methyl-D-aspartate-alpha-amino-3-hydroxy-5-methyl-4-isoxazolepropionic acid (NMDA-AMPA) glutamate neurotransmission cross-talk. The. J. Biol. Chem..

[121] Ma TM (2012). Pathogenic disruption of DISC1-serine racemase binding elicits schizophrenia-like behavior via D-serine depletion. Mol. Psychiatry.

[122] Hikida T (2008). Modulation of d-Serine Levels in Brains of Mice Lacking PICK1. Biol. Psychiatry.

[123] Mustafa AK (2007). Nitric oxide S-nitrosylates serine racemase, mediating feedback inhibition of D-serine formation. Proc. Natl Acad. Sci. U.S.A..

[124] Kolodney G (2015). Nuclear compartmentalization of serine racemase regulates d-serine production: IMPLICATIONS FOR N-METHYL-d-ASPARTATE (NMDA) RECEPTOR ACTIVATION. The. J. Biol. Chem..

[125] Hashimoto A (2007). Effects of MK-801 on the expression of serine racemase and d-amino acid oxidase mRNAs and on the D-serine levels in rat brain. Eur. J. Pharm..

[126] Dumin E (2006). Modulation of D-serine levels via ubiquitin-dependent proteasomal degradation of serine racemase. J. Biol. Chem..

[127] Millar JK (2000). Disruption of two novel genes by a translocation co-segregating with schizophrenia. Hum. Mol. Genet..

[128] Dunlop DS, Neidle A (2005). Regulation of serine racemase activity by amino acids. Brain Res. Mol. Brain Res..

[129] Marchetti M (2013). ATP binding to human serine racemase is cooperative and modulated by glycine. FEBS J..

[130] Betz H (1999). Structure and functions of inhibitory and excitatory glycine receptors. Ann. N. Y Acad. Sci..

[131] Dunlop DS, Neidle A (1997). The origin and turnover of D-serine in brain. Biochem. Biophys. Res. Commun..

[132] Patel AB (2017). Comparison of Glutamate Turnover in Nerve Terminals and Brain Tissue During [1,6-(13)C2] Glucose Metabolism in Anesthetized Rats. Neurochem. Res..

[133] Horiike K (1994). D-amino-acid oxidase is confined to the lower brain stem and cerebellum in rat brain: regional differentiation of astrocytes. Brain Res..

[134] Hashimoto A (1993). Free D-serine, D-aspartate and D-alanine in central nervous system and serum in mutant mice lacking D-amino acid oxidase. Neurosci. Lett..

[135] Sacchi S (2012). Structure-function relationships in human D-amino acid oxidase. Amino Acids.

[136] Strisovsky K (2005). Dual substrate and reaction specificity in mouse serine racemase: identification of high-affinity dicarboxylate substrate and inhibitors and analysis of the beta-eliminase activity. Biochemistry.

[137] Wolosker H (2018). The Neurobiology of d-Serine Signaling. Adv. Pharm..

[138] Berger AJ, Dieudonne S, Ascher P (1998). Glycine uptake governs glycine site occupancy at NMDA receptors of excitatory synapses. J. Neurophysiol..

[139] Rosenberg D (2010). Neuronal release of D-serine: a physiological pathway controlling extracellular D-serine concentration. Faseb J..

[140] Singewald N, Holmes A (2019). Rodent models of impaired fear extinction. Psychopharmacology.

[141] Rosenfield D (2019). Changes in dosing and dose timing of d-cycloserine explain its apparent declining efficacy for augmenting exposure therapy for anxiety-related disorders: an individual participant-data meta-analysis. J. Anxiety Disord..

[142] Miserendino MJ (1990). Blocking of acquisition but not expression of conditioned fear-potentiated startle by NMDA antagonists in the amygdala. Nature.

[143] Wolosker H, Blackshaw S, Snyder SH (1999). Serine racemase: a glial enzyme synthesizing D-serine to regulate glutamate-N-methyl-D-aspartate neurotransmission. Proc. Natl Acad. Sci. U.S.A..

[144] Rodrigues SM, Schafe GE, LeDoux JE (2001). Intra-amygdala blockade of the NR2B subunit of the NMDA receptor disrupts the acquisition but not the expression of fear conditioning. J. Neurosci..

[145] Walker DL (2002). Facilitation of conditioned fear extinction by systemic administration or intra-amygdala infusions of D-cycloserine as assessed with fear-potentiated startle in rats. J. Neurosci..

[146] Ressler KJ (2004). Cognitive enhancers as adjuncts to psychotherapy: use of D-cycloserine in phobic individuals to facilitate extinction of fear. Arch. Gen. Psychiatry.

[147] Rosenberg D (2013). Neuronal D-serine and glycine release via the Asc-1 transporter regulates NMDA receptor-dependent synaptic activity. J. Neurosci..

[148] Lane HY (2013). Add-on treatment of benzoate for schizophrenia: a randomized, double-blind, placebo-controlled trial of D-amino acid oxidase inhibitor. JAMA Psychiatry.

[149] Lin CH (2018). Sodium benzoate, a D-amino acid oxidase inhibitor, added to clozapine for the treatment of schizophrenia: a randomized, double-blind, placebo-controlled Trial. Biol. Psychiatry.

[150] Szilagyi B (2018). Discovery of isatin and 1H-indazol-3-ol derivatives as d-amino acid oxidase (DAAO) inhibitors. Bioorg. Med. Chem..

[151] Graham DL (2019). Human serine racemase: key residues/active site motifs and their relation to enzyme function. Front. Mol. Biosci..

